# The views of postnatal women and midwives on midwives providing contraceptive advice and methods: a mixed method concurrent study

**DOI:** 10.1186/s12884-021-03895-2

**Published:** 2021-06-02

**Authors:** Susan H. Walker, Claire Hooks, Diane Blake

**Affiliations:** 1 Anglia Ruskin University, Bishop Hall Lane, Chelmsford, CM1 1SQ UK; 2 Present address: School of Health & Social Care, London South Bank University, London, UK

**Keywords:** Postnatal contraception, Midwives, Women, Postnatal, UK, Views, Attitudes, Qualitative, Survey

## Abstract

**Background:**

Provision of contraception to women in the immediate postnatal period has been endorsed by professional bodies, to reduce the incidence of short inter-pregnancy intervals. This study examined the views of postnatal women and practising midwives regarding provision of contraceptive advice and contraceptive methods by midwives, in a region of the United Kingdom.

**Methods:**

A mixed-method approach using qualitative interviews with midwives, and a postnatal survey followed by qualitative interviews with postnatal women, in five hospitals in the East of England. Twenty-one practising midwives and ten women were interviewed. Two hundred and twenty-seven women returned a survey.

Survey data was analysed descriptively, augmented by Student’s t-tests and Chi-squared tests to examine associations within the data.

Interviews were recorded, transcribed and analysed guided by the phases of thematic analysis.

**Results:**

Midwives and women supported the concept of increased midwifery provision of contraceptive advice, and provision of contraceptive methods in the postnatal period. Convenience and an established trusting relationship were reasons for preferring midwifery provision over visiting a doctor for contraception.

The best time for detailed discussion was reported to be antenatal and community visits. The Progesterone-only-pill (POP) was the method, in which women indicated most interest postnatally.

Concerns for midwives included the need for increased education on contraceptive methods and training in supplying these. Structural barriers to such provision were time pressures, low prioritisation of contraceptive training and disputes over funding.

**Conclusions:**

Women reported interest in midwives supplying contraceptive methods and expressed the view that this would be convenient and highly acceptable. Midwives are supportive of the concept of providing enhanced contraceptive advice and methods to women in their care, and believe that it would be advantageous for women.

Institutional support is required to overcome structural barriers such as poor access to continuous professional development, and to allow contraceptive provision to be fully recognised as integral to the midwifery role, rather than a marginalised addition.

**Supplementary Information:**

The online version contains supplementary material available at 10.1186/s12884-021-03895-2.

## Background

Women, who have just delivered a baby, are likely to ovulate after 28 days, and are therefore at risk of pregnancy should they resume sexual intercourse [1]. A short inter-pregnancy interval, i.e. fewer than eighteen months from birth to subsequent conception, is detrimental to both mother and baby, and may be prevented by providing women with a reliable form of contraception, which is compatible with breastfeeding, soon after birth [2–5]. Postnatal contraceptive provision from midwives and other professionals has been recommended during the Covid-19 pandemic when access to community services may be limited [6].

Contraceptive advice given during pregnancy and postnatally has been shown to increase the chances of a reliable postnatal contraceptive method being used [7].Women themselves are positive about postnatal provision of contraceptive advice, and methods [8]. Research suggests that although most women intend to use contraception postnatally, they do not appear to receive adequate antenatal or postnatal counselling on postnatal contraception [8–10]. Antenatal counselling is necessary, in addition, to postnatal counselling, to facilitate fully informed consent for immediate insertion post-birth of an intra-uterine device. It is also preferable to postnatal counselling alone for other methods, because the immediate postnatal context may not allow calm, unhurried consideration of contraceptive choices.

Papers reporting postnatal contraception often, but not invariably, assume that the supply of contraceptive long-acting reversible methods (LARC methods)will be provided by members of the obstetric team not midwives e.g. Heller et al. 2017 [10–13]. Midwives in the United Kingdom are an autonomous profession, who are closely involved with women throughout their obstetric journey, and could provide contraceptive methods and contraceptive advice. UK midwifery is transitioning to a ‘continuity of care’ model in which a woman sees the same small team of midwives throughout her antenatal and post-natal journey, which provides a context for repeated discussion of contraception within an ongoing relationship [14]. At present only a small minority of midwives in the UK are trained to fit contraceptive implants or to prescribe contraceptive methods, and these are mostly provided to women who are considered particularly vulnerable e.g. teenagers or those with mental health problems.The World Health Organisation recommends that midwives are trained to fit contraceptive implants, and the Faculty of Sexual and Reproductive Health in the UK supplies training and accreditation for midwives in contraceptive implant insertion [15, 16].

Cameron et al. (2017) report a successful trail of midwifery-provided antenatal contraceptive counselling in the community, but report difficulties arranging for women to receive a LARC method before discharge after birth because of staff workload and time pressures [13]. Croan (2018) reported the successful insertion of a small number of implants by community midwives in the home, as part of the antenatal counselling study by Cameron(2017) [17]. In the USA a survey of midwives found that nine out of ten had never inserted an implant or intrauterine device immediately postnatal, but that half would like the opportunity to do so[18].

The views of postnatal women, and midwives are not well represented in the literature with regard to midwifery – provided contraceptive methods and advice. This study aimed to explore views of midwife-provided contraception from practicing midwives and the women they serve.

## Methods

This was a mixed-methods concurrent study of the views of midwives and postnatal women on the provision of contraceptive advice or methods by midwives. Mixed –methods are suited to health care service research because they allow both a quantitative overview and a more in-depth qualitative exploration of the reasons behind the quantitative responses [19]. Our intention was to understand midwives’ views on contraceptive provision and women’s views on receiving midwifery contraceptive advice/methods. Qualitative interviews were conducted with both women and midwives, supplemented by a quantitative survey of women’s views.

The qualitative interview arm of the study was undertaken in a critical realist paradigm, which assumes a reality expressed by participant’s accounts, but tempered by social experience [20].

### Setting

Five hospitals in the East of England agreed to facilitate the study.

### Midwifery sample (interviews)

Midwives were recruited for interview via a poster displayed in staff areas, and word of mouth. In line with established qualitative sample sizes, and to ensure a variety of settings, we aimed to interview 3–4 midwives from each participating trust for a sample of 20, which is considered adequate for this methodology [20]. Twenty-one midwives were interviewed from five hospitals between June 2019 and March 2020. Written informed consent was obtained before interview and participants received a £15 voucher in recognition of their time.

The interview schedule was designed to explore (a) views on midwives supplying detailed contraceptive advice, advantages of and concerns about this, and views on the best timing of advice, (b) views on midwives supplying contraceptive methods, advantages and concerns about this, (c) which methods should be supplied (d) what training or other changes are needed to allow midwives to supply detailed advice or methods (see Supplementary file 1). Interviews were conducted both face-to-face in the workplace and by phone by three members of the research team (SW, CH, DB), two of whom had a background in midwifery (CH, DB). Interviews were audio-recorded and transcribed before analysis.

### Women’s sample (survey)

The population sampled were women who gave birth in five hospitals in the East of England from June 2019–March 2020. Women who gave birth at home were not included. Women were invited to complete a short survey, and to leave their contact details if they were willing to be interviewed.

The survey was developed for the purpose of the study, and piloted for clarity and acceptability with a small group of women who had recently given birth. The format of the survey was intended to be simple and allow the survey to be rapidly completed by circling answers.The survey consisted of six questions and some demographic information about age, contraceptive method prior to pregnancy, and whether the woman felt her family was complete. The frontpage gave some basic information on contraceptive methods and their suitability postnatally. The first two questions asked about interest in receiving contraceptive advice antenatally or postnatally. These question allowed the responses ‘Yes’or ‘No. The next four questions asked if the women was interested in having each of four methods supplied or fitted by midwives. These were Progesterone-Only Pills (POP), injection, implant and intrauterine methods. The options were ‘Yes, ‘No’ and ‘I am not interested at all in this method’. This last choice was to allow women to indicate if they were uninterested in a method, not simply postnatally but in general. There was also a free-text box to allow women to make any additional remarks.

The inclusion criteria for the survey were intended to allow a sample typical of the usual population of women giving birth within the maternity unit, and so included normal, instrumental and caesarean section deliveries.

Members of the midwifery teams within the five midwifery units distributed a short paper-based survey (See Supplementary file 2) to women, aged 18 years or above, who had given birth, vaginally or by caesarean section. Women were excluded if they were under eighteen years, lacked capacity to consent, could not read and write English sufficiently well to complete the survey unaided, or if the circumstances of pregnancy were such that an approach to complete the survey would cause distress (see Supplementary file 3 for the full protocol and Supplementary file 4 for full exclusion criteria).

Women received the survey in the interval between giving birth and discharge from each hospital. The detachable front page of the survey contained a participant information sheet, including information that informed consent was implied by returning the survey, by posting in a locked collection box situated on the ward. Survey distribution stopped after 3 months or when individual trusts reached a maximum of 50 returned surveys. A total sample of between 150 and 250 women was considered sufficient to allow robust descriptive analysis to be performed, whilst limiting the burden placed on the staff of each trust involved in survey distribution. A formal power calculation was not performed, since this was highly likely to be invalidated by the non-random nature of the sample, but the sample size is similar to previous published surveys [9, 10, 21, 22].

The five hospitals deliver approximately 25,000 women each year so the sample was approximately 1% of women delivering in the five units in the year.

### Women’s Sample (interviews)

Women added their contact details to the survey form, if they were willing to be contacted by telephone for interview. Fifty-seven women left contact details. All were contacted, and those who responded were sent information and a consent form. Ten women returned the form, acknowledging informed consent, and were interviewed by telephone by the lead author (SW). These received a shopping voucher worth £15 as a token of recognition for their time.

The interview schedule was designed to explore four questions, (a) the woman’s contraceptive experience and plans (b) acceptability of receiving contraceptive methods from midwives and how it might be conveniently provided, (c) concerns about receiving contraceptive methods from midwives, (d) best time to receive contraceptive advice (See Supplementary file 5).

### Analysis of interviews

Interviews were recorded, transcribed and analysed guided by the phases of thematic analysis [23]. A semantic approach, aiming to understand the explicit content of the transcripts was applied [24]. Primary codes, relevant to the research aim, were generated from sections of transcript texts, and we searched for patterns in these. These patterns created preliminary themes, which were then merged across transcripts into broader themes. Midwifery and women’s interviews were analysed separately but reported together, since there was substantial agreement between the two samples in terms of the themes arising from the transcriptions.

### Midwifery interviews- analysis

The framework for analysis of midwifery interviews was created by two researchers (SW, CH) analysing the same 4 transcripts and agreeing initial themes, with examples quotations for each of these. The remaining seventeen transcripts were analysed by two researchers (SW, CH) applying the agreed framework, with the option to create new themes if required. These were further refined, to clearly express the views of the participants in relation to midwifery-led contraceptive care. One researcher (SW) then read all 21 transcripts again to ensure that the final themes represented the views expressed in the transcripts, and the significance of the themes was discussed prior to finalising the reporting.

### Women’s interviews- analysis

Qualitative data from women’s interviews was analysed thematically, using both pre-determined and emergent codes, with the aid of NVivo ( NVivo Version 12) software [23]. Pre-determined codes, were derived from the research question and interview schedule, and were focussed on the participant’s attitude to midwifery provision, their experience of receiving contraceptive advice during their care, and their opinion on the optimum timing of giving advice or supplying methods, since these addressed the research topic. The transcripts were read in depth by one researcher (SW), and primary codes relevant to the pre-determined themes and any emerging (and unexpected) codes pertaining to the research were noted. The primary codes were examined, and grouped into broader themes. One new emerging theme was developed. The transcripts were re-read (by CH and SW) to ensure that the four final themes and quotations reflected the raw data. Data saturation was achieved in both samples, indicated by the fact that no new themes were emerging as the final interviews were analysed. Finally the two sets of interviews were integrated to create a coherent picture of the acceptability and feasibility of a midwifery-provided contraceptive service.

### Quantitative survey analysis

Quantitative data was analysed using SPSS (IBM SPSS Statistics for Windows, Version 26). All data was ordinal or categorical, and non-parametric, with the exception of the age of participants, which was continuous and parmetric. Descriptive analysis was augmented by an Independent sample t- tests and Chi-squared tests to examine associations within the data.

#### Data transformation

We created a number of dichotomous variables to facilitate 2 × 2 cross tabulation.

For each individual method we created a dichotomous variable ‘interest in method’ for expressing interest in having the method supplied in the postnatal period i.e. ‘Yes’, and ‘All other responses’ (No, Unsure, Not interested in method, Missed).

We also created a dichotomous variable for ‘pill use prior to pregnancy’ i.e. ‘Pill’ (COCP, POP, pill undefined) versus ‘Other’(all other methods/none).

To determine if a preference for the POP was associated with prior pill use we compared dichotomised contraceptive pill use prior to pregnancy with dichotomous variables reporting interest in being supplied with postnatal POP(as above).

In order to test an association between expressed interest in a method postnatally, and intention to become pregnant in the future, we created a dichotomous variable ‘intention to become pregnant in the future’ using ‘No’ and ‘All other responses’ (Yes, Unsure, Missing) to the question “Do you plan to become pregnant in the future?” We conducted Chi-squared tests of independence for 2 × 2 cross tabulations of dichotomous ‘intention to become pregnant in the future’ and dichotomous ‘interest in method’.

We conducted an independent sample Student’s t-test (2-tailed) to test for association between dichotomised ‘interest in method’ (YES/ all other responses) and age.

### Ethical considerations

This project received ethical approval from the London-Dulwich NHS Research Ethics Committee Ref: 19/LO/0629. This study is listed on the HRA Research Summary site and the study protocol is provided as a Supplementary file (Supplementary File 3).

## Results

Twenty-one midwives were interviewed, with experience ranging from a few months to more than twenty-five years, and drawn from both ward and community teams (See Table 1).

**Table 1:** Demographics of Interviewees

**Participant Code (Midwives)**	**Role**	**Years Qualified**
Midwife A1	Community and hospital	3
Midwife A2	Birthing Unit	22
Midwife A3	Newborn Infant Physical Examination, postnatal clinic	13
Midwife A4	Labour ward	13
Midwife B1	Midwifery -led unit	8
Midwife B2	Ward midwife	4
Midwife B3	Outpatient midwife and Termination of Pregnancy clinic	29
Midwife B4	Delivery suite	5
Midwife C1	Community midwife	30
Midwife C2	Hospital based. Rotational	9
Midwife C3	Safeguarding midwife	34
Midwife C4	Research midwife with experience of other roles	14
Midwife C5	Theatres midwife/ HTU	2
Midwife C6	Research midwife	16
Midwife D1	Central Delivery Suite	5
Midwife D2	Delivery suite	1
Midwife D3	Community Midwife	19
Midwife D4	Foetal medicine midwife/previously labour ward	7
Midwife E1	Teenage midwife	22
Midwife E2	Hospital based	Missing
Midwife E3	Hospital based	4
**Participant Code (Women)**	**Age**	**Family Complete**
Woman A10	23	Not Sure
Woman D1	32	Not Sure
Woman B19	36	Yes
Woman B35	34	No
Woman C14	37	Yes
Woman C16	25	Yes
Woman C26	34	Yes
Woman C31	Missing	Yes
Woman C35	35	Yes
Woman C40	30	No

Ten women were interviewed. All ten had given birth in the previous 6 months. They were using a variety of methods at the time of interview and some had not yet managed to start contraception.

Themes derived from the qualitative interviews are displayed in Table 2:

**Table 2:** Themes derived from the qualitative interviews

Themes	Sub-themes
Role of midwives in contraception at present	
Midwifery provision of contraceptive methods welcomed as quality holistic care	Concerns about midwives providing methods
	Where, when and how contraceptive methods might be provided
Contraceptive advice should be given at multiple points along a journey of midwifery care	Contraceptive advice within an established relationship
	Barriers to giving advice

### Role of midwives in contraception at present

Most midwives reported briefly discussing contraception with women, either on discharge from hospital or during discharge at home. They gave warnings regarding quick return of fertility, and signposted women to their GPs (General Practitioners/family doctors). Midwives who cared for vulnerable women reported that they had more earnest conversations with those women regarding their contraceptive plans.

This was confirmed by the experiences of women who reported that their midwife briefly addressed contraception before discharge or during a home visit, limited to warnings about being fertile quite soon after birth, and signposting to the woman’s family doctor. Some women reported being given leaflets or watching a DVD on contraception before discharge, but did not find these particularly helpful. Most women felt that the issue of contraception was addressed in a superficial or hurried manner, which one described as a *‘like a tick box’***Woman B19**.

### Midwifery provision of contraceptive methods as part of quality holistic care

Midwives had positive views about the proposed role of midwives in provision of contraceptive methods. They felt that this was part of a midwife’s role in terms of health promotion, and fitted into the concept of continuity of care. They expressed the view that making access to contraception easier would have benefits for women and argued that this was particularly important for vulnerable women and their children. Midwives understood that the need to make an appointment with a doctor after six weeks to receive contraception was a hazardous discontinuity of care, which could result in unintended pregnancy.

*“Well, as a midwife, I think it's a good thing because you're giving holistic care. If you see the women at the beginning of their pregnancy until the end, before they're discharged, or if they are in the hospital or if it's in the community, they don't need to wait for another six weeks.”* **Midwife C6.**

*“For the women, I think the fact that it’s sorted. You’re less likely to get the surprise pregnancies, as such, when they come back into us within a year. Because sometimes, not seeing your GP until six to eight weeks can be too late”* **Midwife B2.**

There was enthusiasm for midwives learning and undertaking the training of younger midwives, thus cascading training through peer teaching.

*“The problem is going to be to educate, because that is going to cost some amount of money. It’s time as well, and, obviously, our [Practice Development] midwives would need to be able to facilitate that to our colleagues[…] So, I think that that would be a little bit tricky initially. But, once you’ve got a number of midwives who are competent and happy… then the ball is just rolling, isn’t it? It’s following and then the students can do it.”* **Midwife E2.**

Women who were interviewed unanimously welcomed midwifery involvement in supplying contraceptive methods, and did not distinguish between prescription of a pill method or supplying an injection, implant or intrauterine device. They viewed midwifery provision as more convenient and comfortable than making an appointment with a doctor.

*‘No, I think it was just how strongly I feel about it and how good I think it would be for women. I think it may be more comforting for some women than having to go to the doctors, waiting for a nurse and stuff. Whereas if your midwife does it you know them.’* **Woman A10.**

Several women expressed the view that midwives were more knowledgeable about the interaction of contraception and breastfeeding than their GP, and were therefore ideal people to be involved in discussing contraception.

*‘Because I was breastfeeding, as well, he didn’t know what I could take, so he had to look it up, and midwives might have a bit more of a clue of whether you're allowed to take it or not, and things like that.’* **Woman D1.**

Conceptually midwives separated the supply of pills or the contraceptive injection, from the fitting of implants or intra-uterine devices, when discussing the feasibility of supplying methods. Some midwives were cautious about becoming involved in intra-uterine contraception, lacked experience of and knowledge about the practice, and felt it was best left to medical staff.

#### Subtheme; concerns about midwives supplying contraceptive methods

Midwives recognised that not all of their colleagues might feel positively towards the concept of enhanced contraceptive provision by midwives. There was anxiety about the midwifery role being already very full and concern that providing an extra service to women would come at the cost of increased midwifery workload, at a time when midwives were already stretched and under pressure.

*“I think our role at the moment is so broad and we’re so stretched, I think it would just put an extra strain on the service that I don’t think we’d be able to cope with it.”* **Midwife B4.**

Midwifery participants expressed the need for support to learn and practice new skills, and the need for more education and training. They were doubtful that training to provide contraception would have priority in terms of funding, and protected time to learn.

There were concerns expressed about the pathways involved in funding and anticipation that wrangles over budgeting might prevent midwifery involvement in contraceptive supply.

*“Who is going to fund it? Is it going to be the GP? Is that a primary care responsibility or is it an obstetric responsibility? It comes under women and child health but is it in the primary sector or is it in the hospital sector. That's a barrier to it, isn't it?”* **Midwife A3.**

In contrast, women expressed few concerns about midwifery provision of contraception, provided midwives were appropriately trained. Concurring with midwives, the most common concern was about midwives’ heavy workloads resulting in not having time for contraceptive discussion, which women did not want to become a rushed or token exercise.

#### Subtheme: when, where and how contraceptive methods might be provided by midwives

Midwives expressed concern that an extended contraceptive role for midwives, involving supplying contraceptive methods, should be properly structured and incorporated into the formal pathway of care. It was seen as important that midwifery expertise is not based in one single person, and so lost during periods of annual leave or similar absence, but that a team of midwives should develop expertise in supplying contraception, and/or that every midwife should develop more skills and knowledge in this area. It was suggested that midwifery provision could become a specialist role, similar to examination of the new-born, thus removing the extra task from midwives in general, but there was also some concern that specialising in this respect would deplete the ward staffing.

Midwives were not convinced that the immediate postnatal ward setting was an appropriate time to supply contraception, because of time pressure, and the desire of women to get home quickly.

*“I think immediately after… I really don’t know, but, honestly, our delivery unit goes like a train most of time and women are already having to wait for suturing and things like that. I wonder if that would just be one thing too many, but that’s my immediate reaction.”* **Midwife C3.**

Community midwives were viewed as well placed to provide an enhanced contraceptive service because they saw women out of a busy hospital environment, and at a stage when women might welcome the intervention. GP surgeries, or a midwifery–led clinic based in hospital premises, were suggested as alternative venues for midwives to provide contraception requiring a clinical environment, with the added advantage of prolonged contact with the midwife.

*“But then again, our community midwives obviously work in GP’s surgeries and they’ve got the clinical room, so that’s another place. But, that’s if they ladies want and are going to be willing to go. I’m thinking most ladies probably are going to, because they’re going to see their midwife again. Even if that is not on day 10, we could extend it to day 15 or day 20. They are going to see the midwife, they will have an opportunity to speak to the midwife, talk about the baby and maybe there will be some other issues.”* **Midwife E2.**

In general, women also viewed midwifery supplied contraception as convenient, whereas arranging to see a doctor for contraception was difficult and stressful. Like midwives, some were in favour of a midwifery-led contraceptive clinic.

*‘It would almost be good if you could have drop-in sessions [for midwifery-led contraception]. If it wasn’t so intense and it was an appointment- Like you knew it was there and you could go. If you didn’t get around to going the first week, because your baby sicked up or something […] In those first weeks, everything seems like such an effort and you feel like you’re never going to get out of the house’* **Woman C40.**

### Contraceptive advice should be given along a journey of midwifery care

Women and midwives were unsure about the best time for giving contraceptive advice, with arguments made for ante-natal, immediate postnatal and delayed postnatal discussion. This is also demonstrated in the survey responses (See Table 4).

Midwives reported that addressing the subject of contraception in the antenatal setting was desirable, particularly since other discussions about postnatal decisions, e.g. breastfeeding, take place at this time. However there was also concern that time was limited in an antenatal setting, and that women may not be focussed on contraception. There was general agreement that the antenatal setting was a time to *“plant the seed”* (**Midwife A2**) in regard to contraception, which would allow more extended discussions with women who expressed interest at a later date.

After birth and before discharge from community midwifery care, either at Day 10 discharge or a little later, was viewed by both midwives and women as a good time to have a detailed discussion about contraception.

*“I think probably the best time would either be before the birth or ten days after, like a few days after. Cos, just from my experience I was so exhausted and overwhelmed during those first few days after her being born. Like trying to process that was a bit would have been a bit too much.”* **Woman E18.**

*“I think it's very different in the community because you've allowed that family to have a little bit of time to get over the birth. Then at the end of your week, 10 days, then you feel like you're in a bit more of a position to be able to have those conversations with women.”* **Midwife A3.**

#### Subtheme: contraceptive advice within an established, trusting relationship

Midwives and women reported that midwives were well placed to provide contraceptive advice because they had an established relationship with women, and women felt comfortable talking to them about intimate issues.

*“I do think that the advantage probably is that for most women they really trust their midwives. They trust our opinion. They trust our experience. A lot of women we've built up quite a nice relationship with them. I think we're in a really privileged position really, aren't we, going into their homes and chatting with them. Certainly when you've got your own caseload and you really know the women, you can bring it up very, very easily.”* **Midwife C1.**

*“With midwives like I’m finding like they’re probably the best people to go to… I’d prefer it, to be fair, to have a midwife rather than a doctor […] They know what it’s like and I can relate more, they’ll discuss things with me and have more time for me, and I don’t feel GPs [ i.e. doctors] do.”* **Woman C31.**

#### Subtheme: barriers to giving advice – time, privacy and knowledge

Midwives identified, as a barrier to giving more detailed advice, the fact that low risk women were often discharged very quickly from the ward and had other priorities. They also reported concerns about finding time to give detailed advice properly.

Our survey also found that about one third of women were not interested in midwives supplying advice on contraception either ante-natally or postnatally. Where women supplied reasons for not wanting advice, in the free text comments, they indicated that midwives were too busy, or that the postnatal period was too busy for the woman herself, with too much information to take in.

Midwives reported that privacy was also a problem in busy wards, although it was felt that birthing-units with private rooms were more conducive, and that a private space could be found on a ward if required. Time alone with the woman and her partner was sometimes difficult to find.

*“Well, in the birthing centre they have their own rooms. The only thing is if the husband is there. If the husband is there, it's much better because they can both decide for themselves. The only thing is there are so many relatives in there, so you need to find time when there are no people around, the relatives aren't visiting.”* **Midwife C6.**

In contrast to women’s reports of feeling confident about midwifery knowledge of contraception, midwives reported that they lacked the knowledge to feel confident to have more detailed contraceptive discussions, and were unsure about when and which methods could be started safely postnatally. Few UK-trained midwives interviewed had received any formal education on contraception since registration, but midwives who had trained abroad reported more contraceptive education. On a more immediately practical level, midwives expressed the need for comprehensive and up-to-date leaflets on contraception, which could be given to women, but could also guide the midwifery conversation, so that midwives were sure that they were giving the correct information.

*“I think I'd need to come from a place where I feel confident about the information that I'm giving women and being able to practice what is expected of me. So that will come from, hopefully, some place of learning.”* **Midwife B1.**

#### Quantitative findings

Two hundred and twenty seven women returned the survey (see Table 3). Response rates for the survey are available for only three of the five hospitals taking part and range from 25–40%. Free text comments from the survey concurred with the views expressed by the women who were interviewed.

**Table 3:** Demographics of Survey Respondents *N* = 227

**Age**	Range (19–46), Median = 32, Mean = 31
**Method prior to pregnancy**	% (n)
None	22.9 (52)
COCP, POP + Pill (undifferentiated)	39.2 (89)
Condom	15.9 (36)
Injection	2.6 (6)
Implant	2.6 (6)
IUD, IUS + IUC (undifferentiated)	5.7 (13)
Other method e.g. Persona	3.5 (8)
More than one method reported	2.2 (5)
Missed Response	5.3 (12)
*Total*	*100 (227)*
**Intends future pregnancy**	
Yes	37.9 (86)
No	33.9 (77)
Not Sure	26 (59)
Missed	2.2 (5)
*Total*	*100 (227)*

The method in which women responding to the survey indicated most interest was the progesterone-only –pill (47%) (see Table 4). Longer acting methods were of less interest but approximately 20% of women indicated interest in either an implant or an intra-uterine device.

**Table 4:** Interest in receiving specific contraceptive methods from midwives

**Interested in a specific method supplied by midwives**	**YES** **% (n)**	**NO** **% (n)**	Not interested at all in method % (n)	Unsure % (n)	Missed % (n)	*Total* *% (n)*
POP	47.1 (107)	29.5 (67)	21.1 (48)	0.4 (1)	1.8 ( 4)	*100 (227)*
Injection	32.6 (74)	41.0 (93)	25.6 (58)	0	0.9 (2)	*100 (227)*
Implant	19.4 (44)	46.7 (106)	33.0 (75)	0	0.9 (2)	*100 (227)*
Intrauterine Contraception	20.3 (46)	52.0 (118)	26.4 (60)	0	1.3 (3)	*100 (227)*

Women who had taken a contraceptive pill before pregnancy were much more likely to express interest in midwives supplying a pill postnatally.

Chi-squared tests showed a highly significant association between prior pill use (COCP and POP, pill undefined) and interest in postnatal POP prescription (*x*^2^(1,*N* = 227) = 16.797, *p* < 0.001 (2-sided)). No association with interest in other methods was observed.

Interest in specific methods was independent of age, but women who indicated that their family was complete were more likely to indicate interest in an intrauterine method from midwives. Chi-squared tests showed a statistically significant association with interest in having an IUC fitted by midwives, and having no plans for a future pregnancy (*x*^2^(1,*N* = 227) = 6.654, *p* = 0.010 (2-sided)).

A majority of women responding to the survey reported interest in receiving contraceptive advice from midwives, during pregnancy (56%) and postnatally (63%), although approximately 30% of women indicated that they did not wish to receive advice (see Table 5).

**Table 5:** Responses to interest in receiving advice

**Interested in receiving contraceptive advice**	**YES** **% (n)**	**NO** **% (n)**	Missed % (n)	*Total* *% (n)*
- Antenatally	56.4 (128)	31.7 (72)	11.9 (27)	*100 (227)*
- Postnatally	62.6 (142)	30.4 (69)	7.0 (16)	*100 (227)*

## Discussion

### Limitations and strengths

This study is timely, in the context both of a pandemic which limits access to community contraceptive services, and the concern about fragmentation and reduction in contraceptive services which has occurred in the last 10 years [25–27]. It has employed a rigorous approach to data collection and analysis. Our choice of participants was guided by the aim of learning the opinions of direct stakeholders in postnatal contraceptive provision, which is to say midwives, who are and will be actually involved in supplying contraceptive advice or methods, and women who have just given birth [28]. By including women regardless of method of delivery, this study sampled a wide variety of women, typical of those having their baby in hospital setting. In drawing on the views of midwives and women from five different hospitals within a geographic region, it has used a sample both varied and homogenous enough to be transferable to other similar settings [28].

A limitation of the study is the self-selected sampling method, which will have introduced bias, since midwives and women most interested in contraception will have volunteered to take part. Response rates for the survey are available for three of the five hospital sites and range from 25–40%, indicating that many women declined to return the survey. The lack of formal response rates for two sites is a limitation.

Midwives and women who chose to take part are most likely to have an interest in participating in midwifery-led contraception, adding authenticity and credibility to their views on how it might be done. Conversely midwives and women who do not have any interest in providing or receiving contraceptive care may have chosen not to take part, and so the results of this study may not fully reflect the barriers to implementing midwife-led contraceptive services.. The small and non-random sample of women completing the survey limits the generalisability of its findings, and the robustness of inferential analysis carried out.

#### Context of previous research

Our midwifery participants felt that they should be involved in contraceptive provision but needed training and institutional support to do so. They were best placed to provide contraception but not best equipped. The lack of confidence in contraceptive knowledge and need for further training in contraceptive methods is in line with previous research [12, 29–31]. Women reported not finding information leaflets on contraception particularly helpful, and preferred a face-to-face conversation, whereas some midwives suggested the use of leaflets to ensure that they were giving the correct information. This suggests that there is a role for contraception information resources in the form of leaflets, but that these should be used to support, not replace face-to-face tailored advice.

The lack of time, and concerns about an expanding remit have been previously reported, as have concerns about the unsuitability of the immediate postnatal period for detailed discussion [30, 32].

Our findings concur with previous surveys of antenatal and postnatal women in the UK, Italy and US, in finding that most women both needed and welcomed contraceptive advice antenatally and postnatally, and that most viewed favourably the provision of postnatal contraception before hospital discharge [9, 10, 12, 13, 21, 33]*.* Cameron et al. (2017) surveyed 794 UK women taking part in a trial of antenatal midwifery-led contraceptive advice, and report that 74% found this advice helpful [13].

Di Giacomo et al. report that only 45% of women in a cross-sectional survey of women discharged from a maternity ward had received adequate information about contraception, and lacked knowledge about which contraception to use when breastfeeding and about return of fertility after birth [21]. The authors note that midwives are well placed to supply accurate information. Weisband et al. (2017) in the US surveying 100 breastfeeding before discharge, similarly found that only 57% had discussed contraception with a prenatal care provider, and 21% had not considered the effects of contraception upon breastfeeding [9] Thwaites & Bacon (2016) report a survey of 112 women in a UK hospital, of which 84% would have liked to receive contraception or contraceptive information on the post-natal ward [10]. A subsequent survey of 272 women in a UK postnatal ward indicated that 32% women needed more information about postnatal contraception and that 47% would prefer to receive their method before leaving the ward [8].

Carr et al. report that convenience was a key motivator in women choosing immediate postnatal contraception and the women interviewed in our study also valued highly the potential convenience of midwifery supplied, postnatal contraception [34].

Our paper fills a gap in the evidence regarding the views of women on receiving contraceptive methods and/or advice from their midwives during their maternity care, by providing details of which methods women were interested in receiving from their midwives. It also provides qualitative detail about women’s reasons for their views.

The interview analysis found that women had difficulty attending doctor’s appointments after having a baby, and sometimes felt that family doctors lacked knowledge of the effects of contraceptive methods on breastfeeding. Women expressed trust in midwives’ knowledge of their and their babies’ health, and reported finding it easy to talk to midwives about intimate concerns.

Both survey and qualitative data reflected division of views on the ideal timing of midwifery-provided contraceptive advice. This suggests that contraceptive discussions should take place at several stages along the pathway of midwifery care – antenatally, immediately postnatally and in the community. Midwives suggested that discussions about contraception supply after delivery should be introduced into the antenatal care pathway, allowing preferences about contraception to be recorded in a women’s notes, allowing it to be re-visited after delivery by ward-based or community midwives. They emphasised that antenatal midwives should be trained and supplied with support material, to allow reasonably detailed discussions to take place. This approach has been demonstrated successfully previously with community midwives embedding contraceptive discussion at 22 weeks gestation into their care [13].

Complementing previous studies showing that postnatal fitting of LARC methods is well accepted and effective in preventing short IPI, women who responded to our survey in the UK, where all methods are free of charge, were most enthusiastic about shorter-acting options [12, 34–40]. The method in which women expressed most interest was progesterone-only pills (POP). This may be due to familiarity with the method, since 39% of women who answered the survey were using a pill prior to pregnancy and these women were statistically more likely to indicate interest in receiving a pill postnatally. Qualitative interviews suggested that some women felt that long-acting methods were too ‘permanent’ a choice to make, especially if not considered beforehand. It has been previously reported that women are unaware that LARC methods need not be retained for the full duration of their effectiveness, and this may influence women’s views on postnatal LARC fitting [41]. This suggests that midwifery provision of contraception should offer shorter-acting methods such as the POP or injection, as well as longer-acting methods.

### Implications for practice and policy

Most women are interested in receiving contraceptive advice from midwives, and policy makers and practitioners should consider how this can be meaningfully built in to the usual structure of midwifery care, to avoid time constraints resulting in a tokenistic ‘tick-box’ approach. The term ‘advice’, as used by participants in our interviews, can cover a range of interactions from simple signposting to other services, through to detailed advice on various methods. We suggest that midwives should aim to provide high quality contraceptive counselling, as described by Dehlendorf [42], which optimises information-giving on all aspects of contraception and facilitates informed decision-making, within an atmosphere of mutual respect and trust. This will require contraceptive education during midwifery pre-registration training, and opportunities to keep up-to-date through professional development. The Nursing & Midwifery Council (NMC) in the UK is considering how to revise midwifery training standards, which might present an opportunity for greater emphasis on contraception. An apprenticeship model, where more experienced midwives cascade training during their mentorship of junior colleagues, was suggested by one of our interviewees. The lack of mentor supplied modelling around contraceptive advice-giving has been noted in previous research, and may be an area were strengthening of the skills base of qualified midwives could enable and encourage midwifery students to develop their knowledge [29].

Beyond reiterating barriers to enhanced contraceptive provision, this project has provided an outline of how practising midwives and women feel contraceptive services could be provided in their area of practice and the factors that will need to be in place to allow this to be sustainable.

In terms of midwives supplying contraception, the method in which women expressed most interest was the POP. Arguably this should be the focus of general midwifery provision, either as a long-term method, or a bridging method until LARC provision can be arranged. Some women would welcome the opportunity to have LARC methods fitted by specialist midwives, either immediately post-delivery or before discharge from care. Midwifery interviewees felt that community midwives could administer contraceptive injections or supply progesterone only pills within the first three weeks after delivery, thus avoiding the time constraints of supplying these methods before discharge in a postnatal ward.

Contraceptive advice and provision by midwives requires management investment to overcome structural barriers, such as lack of clarity over who pays for methods that midwives provide. Policy makers should consider how the training of midwives can be enhanced to allow them to prescribe and/or fit contraceptive methods. Steps in this direction are already being undertaken in the UK context [16]. Funding for the training of adequate numbers of midwives to prescribe or administer contraception is needed so that a team-based approach can be put in place to ensure consistency of services.

The opportunity for antenatal discussion and postnatal provision of contraception needs to be embedded in the structure of routine antenatal and postnatal care, incorporated into routine paperwork and recognised in terms of staff rotas and allocated patient time. This requires strong management support.

The need for strong management ‘buy-in’ and multi-disciplinary cooperation across professional, and primary and secondary care boundaries has been noted previously, mostly in regard to LARC provision [12].

## Conclusion

This study suggests that women in midwifery care in the UK feel they would benefit from advice from midwives on contraceptive methods, and on their suitability when breastfeeding. This should be provided at multiple times along the care pathway, antenatally and postnatally, because women report different views on when they would be receptive to discussing contraception.

Women also indicated interest in midwives supplying contraceptive methods, viewing this as convenient and highly acceptable.

Our participants, who were practising midwives, were enthusiastic about enhanced midwifery provision of contraception, because they saw it as beneficial for women, and part of their holistic approach to care. They favoured the provision of contraceptive advice and methods, not in a hurried, one-time exercise immediately after birth, but as series of discussions and decisions. However important barriers to midwifery involvement need to be addressed at a managerial and institutional level.

## Supplementary Information

**Additional file 1.**

**Additional file 2.**

**Additional file 3.**

**Additional file 4.**

**Additional file 5.**

**Additional file 6.**

## Data Availability

The datasets generated and/or analysed during the current study are not publicly available due because consent for this was not obtained from participants. Restricted anonymised survey data may be available from the corresponding author on reasonable request.

## Abbreviations

IPI: Inter-pregnancy Interval
POP: Progesterone Only Pill
LARC: Long acting reversible contraceptives
GP: General Practitioner (family doctor)
NMC: Nursing & Midwifery Council
UK: United Kingdom
