# Supplementary material for: The views of postnatal women and midwives on midwives providing contraceptive advice and methods: a mixed method concurrent study
Source: BMC Pregnancy Childbirth. 2021 Jun 2;21:411. doi: 10.1186/s12884-021-03895-2 (PMC8170056; doi:10.1186/s12884-021-03895-2)
Supplement: Supplementary file 1 — Additional file 1. [file 12884_2021_3895_MOESM1_ESM.docx]

Draft Interview schedule for Midwifery staff

Check consent

Demographics – role, how long in post, previous contraceptive training

What are your views on midwifery staff providing detailed advice on contraception?

When would be the best time for this to take place?

What would be the advantages of it for women? For midwives?

Would you have any concerns about this?

What would be helpful for you to allow this to take place? Training? Tools? Structure? Indemnity?

What are your views on midwifery staff providing contraceptive methods?

Which methods do you think they should supply?

For each method:

When would be the best time for this to take place?

What would be the advantages of it for women? For midwives?

Would you have any concerns about this?

What are the problems with this?

What would be helpful for you to allow this to take place? Training? Tools? Structure? Indemnity?

Any other views or comments?
