# Supplementary material for: The views of postnatal women and midwives on midwives providing contraceptive advice and methods: a mixed method concurrent study
Source: BMC Pregnancy Childbirth. 2021 Jun 2;21:411. doi: 10.1186/s12884-021-03895-2 (PMC8170056; doi:10.1186/s12884-021-03895-2)
Supplement: Supplementary file 2 — Additional file 2. [file 12884_2021_3895_MOESM2_ESM.docx]

| Brief Information on Contraception after Childbirth | |
| --- | --- |
| Sexual intercourse as soon as 21 days after giving birth can result in pregnancy.  A woman who does not want to get pregnant needs to start a method of contraception by the time her baby is 21 days old. | 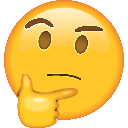 |
| All except one of the commonly used methods of contraception are safe when breastfeeding, and can be started soon after giving birth, provided mother and baby are well. | 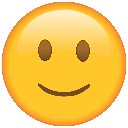 |
| The exception is the combined oral contraceptive pill (COCP) which must not be started until the baby is 6 weeks old, if breastfeeding, or 3 weeks old if not. | X |
| Progesterone-only pills (POP) are safe for the mother, safe when breast-feeding, and can be started as soon as needed after giving birth. | 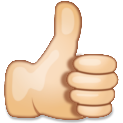 |
| Contraceptive injections are safe for the mother, safe when breastfeeding, and can be started as soon as needed after giving birth. The injections last for 3 months. | 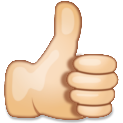 |
| Contraceptive implants are safe for the mother, safe when breast feeding, and can be implanted as soon as convenient after giving birth. Each implant can last for 3 years. | 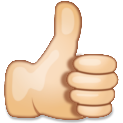 |
| Intrauterine devices (‘copper coils’) and intrauterine systems (‘hormonal coils’) are safe if inserted within 48 hours of childbirth, and safe when breast-feeding. If this 48- hour window is missed insertion must be delayed until four weeks after giving birth. | 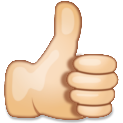 |

You can detach this page if you would like to keep the information.

Please answer the questions on the **next page** and return the survey to your midwife or to us.

If you prefer you can answer the survey on your phone or online at <https://angliaruskin.onlinesurveys.ac.uk/midwifery-contraceptive-provision>

Or [**https://tinyurl.com/y88djmqy**](https://tinyurl.com/y88djmqy)

The questions below are asking your opinion on whether you would be interested in having the following contraceptive services supplied by your midwife, immediately after childbirth, assuming they were safe and appropriate for you.

You can skip any questions that you do not want to answer.

Please circle your answer e.g.

**Q1. I would be interested in having detailed and specific advice on contraceptive methods from my midwife:**

| YES | NO |
| --- | --- |

- During my antenatal care.

| YES | NO |
| --- | --- |

- In the few days after my baby is born.

| YES | NO | I am not interested at all in this method |
| --- | --- | --- |

**Q2. If available, I would be interested in having progesterone-only contraceptive pills prescribed for me by my midwife, in the few days after my baby is born.**

| YES | NO | I am not interested at all in this method |
| --- | --- | --- |

**Q3. If available, I would be interested in having a contraceptive injection from my midwife in the few days after my baby is born.**

**Q4. If available, I would be interested in having a contraceptive implant fitted by my midwife in the few days after my baby is born.**

| YES | NO | I am not interested at all in this method |
| --- | --- | --- |

**Q5. If available, I would be interested in having an intrauterine device (copper coil) or intrauterine system (hormonal coil) fitted by my midwife in the first 48 hours after giving birth.**

| YES | NO | I am not interested at all in these methods |
| --- | --- | --- |

**Please add any comments in the box below.**

**Details about you:**

- What age are you?
- What method of contraception were you using before becoming pregnant?

| YES | NO | NOT SURE |
| --- | --- | --- |

- Do you hope or plan to become pregnant again in the future?

Thank you for completing this survey.

If you consent to be included in this research project, please hand this questionnaire to your midwife or post it in the secure locked postbox on the ward, or return it online [**https://tinyurl.com/y88djmqy**](https://tinyurl.com/y88djmqy).

If you do not wish to take part you do not have to return this survey.


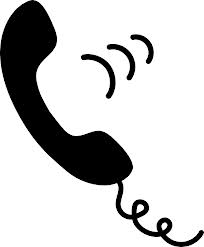
We would also like to interview some post-natal women to hear their views on this topic. The interviews will take place by telephone in the next few weeks. Each person interviewed will receive a £15 shopping voucher for their time.

If you think you might be interested in taking part in a telephone interview, and sharing your views, please put you name and email address or telephone contact details here.

A researcher will contact you with more information to help you decide whether to take part further.

If you are not interested in being interviewed this is the end of your participation in the project. Please return the survey.

Thank you very much for taking part.
