# Supplementary material for: The views of postnatal women and midwives on midwives providing contraceptive advice and methods: a mixed method concurrent study
Source: BMC Pregnancy Childbirth. 2021 Jun 2;21:411. doi: 10.1186/s12884-021-03895-2 (PMC8170056; doi:10.1186/s12884-021-03895-2)
Supplement: Supplementary file 3 — Additional file 3. [file 12884_2021_3895_MOESM3_ESM.docx]

**Qualitative Protocol Development Tool**

The research protocol forms an essential part of a research project. It is a full description of the research study and will act as a ‘manual’ for members of the research team to ensure adherence to the methods outlined. As the study gets underway, it can then be used to monitor the study’s progress and evaluate its outcomes.

The protocol should go into as much detail about the research project as possible, to enable the review bodies to fully understand your study.

The use of this collated consensus guidance and template is not mandatory. The guidance and template are published as standards to encourage and enable responsible research.

The document will:

- Support researchers developing protocols where the sponsor does not already use a template
- Support sponsors wishing to develop template protocols in line with national guidance
- Support sponsors to review their existing protocol template to ensure that it is in line with national guidance.

A protocol which contains all the elements that review bodies consider is less likely to be delayed during the review process because there will be less likelihood that the review body will require clarification from the applicant.

We would appreciate self-declaration of how you’ve used this template so we are able to measure its uptake.

Please indicate the compatibility of this template with any existing templates you already use by stating one of the following on the front of each submitted protocol:

- **This protocol has regard for the HRA guidance and order of content**

**FULL/LONG TITLE OF THE STUDY**

Feasibility and acceptability of the provision of contraceptive advice and treatment by midwives to women in their care

**SHORT STUDY TITLE / ACRONYM**

Provision of contraceptive advice and treatment by midwives

**PROTOCOL VERSION NUMBER AND DATE**

**1.0 31-01-19**

**RESEARCH REFERENCE NUMBERS**

| **IRAS Number:** | 252063 |
| --- | --- |
| **SPONSORS Number:** | N/A |
| **FUNDERS Number:** | N/A |

**This protocol has regard for the HRA guidance and order of content**

# SIGNATURE PAGE

The undersigned confirm that the following protocol has been agreed and accepted and that the Chief Investigator agrees to conduct the study in compliance with the approved protocol and will adhere to the principles outlined in the Declaration of Helsinki, the Sponsor’s SOPs, and other regulatory requirement.

I agree to ensure that the confidential information contained in this document will not be used for any other purpose other than the evaluation or conduct of the investigation without the prior written consent of the Sponsor

I also confirm that I will make the findings of the study publically available through publication or other dissemination tools without any unnecessary delay and that an honest accurate and transparent account of the study will be given; and that any discrepancies from the study as planned in this protocol will be explained.

| **For and on behalf of the Study Sponsor:** | | |
| --- | --- | --- |
| Signature:  ..... 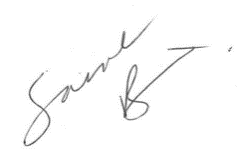..................................................................... |  | Date: .8...../.2...../.19..... |
| Name (please print):  ......Sarah Burch.......................................................................... |  |  |
| Position: .......Director of Research.............................................. |  |  |
| **Chief Investigator:** | | |
| Signature: 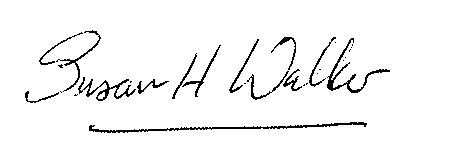...................................................................................................... |  | Date: .4th../.Feb./19...... |
| Name: (please print):  Susan Walker................................................................ |  |  |

#

# LIST of CONTENTS

| **GENERAL INFORMATION** | **Page No.** |
| --- | --- |
| HRA PROTOCOL COMPLIANCE DECLARATION | i |
| TITLE PAGE | ii |
| RESEARCH REFERENCE NUMBERS | ii |
| SIGNATURE PAGE | iii |
| LIST OF CONTENTS | iv |
| KEY STUDY CONTACTS | v |
| STUDY SUMMARY | v |
| FUNDING | vi |
| ROLE OF SPONSOR AND FUNDER | vi |
| ROLES & RESPONSIBILITIES OF STUDY STEERING GROUPS AND INDIVIDUALS | vi |
| STUDY FLOW CHART | vii |
| SECTION | |
| 1. BACKGROUND | 1 |
| 2. RATIONALE |  |
| 4. RESEARCH QUESTION/AIM(S) |  |
| 5. STUDY DESIGN/METHODS |  |
| 6. STUDY SETTING |  |
| 7. SAMPLE AND RECRUITMENT |  |
| 8. ETHICAL AND REGULATORY COMPLIANCE |  |
| 9. DISSEMINATION POLICY |  |
| 10. REFERENCES |  |
| 11. APPENDICES |  |

# KEY STUDY CONTACTS

| Chief Investigator | Susan Walker, Anglia Ruskin University, [susan.walker@anglia.ac.uk](mailto:susan.walker@anglia.ac.uk); 01245 684663 |
| --- | --- |
| Sponsor | Anglia Ruskin University, Sarah Burch, 205, Young St, Cambridge. [Sarah.burch@anglia.ac.uk](mailto:Sarah.burch@anglia.ac.uk). Tel: 01223 698560 |
| Funder(s) | Health Education England (East of England) |

**STUDY SUMMARY**

| Study Title | Feasibility and acceptability of the provision of contraceptive advice and treatment by midwives to women in their care |
| --- | --- |
| Internal ref. no. (or short title) | Provision of contraceptive advice and treatment by midwives |
| Study Design | Qualitative with descriptive quantitative survey |
| Study Participants | Women receiving post-natal care in 7 acute trusts  Midwives providing post-natal care in 7 acute trust |
| Planned Size of Sample (if applicable) | Total 464  Max 64 qualitative interviewees  Max 400 survey respondents |
| Planned Study Period | April 2019- June 2020 |
| Research Question/Aim(s) | 1. To what extent are post-natal women supportive of the idea of an enhanced provision of contraception by midwives in the post-natal period? What is the extent of interest in particular groups, in terms of age, and pregnancy intention, or contraceptive use before pregnancy? 2. What are the views, concerns, perceived benefits, and reported needs of women with regard to the enhanced provision of contraception by midwives in the post-natal period? 3. What are the perceived benefits, concerns, logistical barriers, and training needs expressed by midwives with regard to the feasibility of enhanced provision of contraception by midwives in the post-natal period? |

**FUNDING AND SUPPORT IN KIND**

| **FUNDER(S)**  (Names and contact details of ALL organisations providing funding and/or support in kind for this study) | **FINANCIAL AND NON FINANCIALSUPPORT GIVEN** |
| --- | --- |
| **Health Education England (East of England)** | **Funding for design, management and conduct of project** |

**ROLE OF STUDY SPONSOR AND FUNDER**

The funder approved the research aims but has had no influence of the design and conduct of the research.

| **KEY WORDS:** |  |
| --- | --- |

Postnatal

Contraception

Midwives

UK

Attitudes

# STUDY FLOW CHART

| Nov 18 – March 19 | - Seeking gatekeeper permission - Preparation of surveys and interview schedules - Completing HRA application for ethical approval and Research Governance approval. - Application for research passports and honorary contracts, where required, from research team |
| --- | --- |
| April 19 | - Start of data gathering, start of recruitment phase |
| Mar 20 | - Completion of data gathering, end of recruitment phase - Start of analysis and interim write up |
| June 20 | - Completion of analysis - Submission of paper or report - Start of dissemination of findings |

Scheme of data collection in each trust

HoM interview

Trust 1-7

Interview x 4 (max) midwives

**STUDY PROTOCOL**

Feasibility and acceptability of the provision of contraceptive advice and treatment by midwives to women in their care

# 1 BACKGROUND

The giving of advice on contraception and the planning of future pregnancies is part of the midwifery role and competencies in the UK and Europe. The European Union Directive Recognition of Professional Qualifications 2005/36/EC Article 40 makes it incumbent upon member states to ensure that midwives are able to, at the point of registration, “provide sound family planning information and advice” (1). NICE guidelines on post-natal care state “Methods and timing of resumption of contraception should be discussed within the first week of the birth” (2)

In the last 50 years, the range of contraception available to women has increased and most forms of contraception are suitable for use in the post-natal period, and during breast-feeding. In particular, contraceptive implants are safe, highly effective, long-acting and do not affect breast milk production. With the development of non-medical prescribing, and the training of non-medical clinicians (mostly nurses working in contraception and sexual health settings) to fit contraceptive implants, midwives are well placed to advise women on contraceptive methods, to prescribe these methods and to fit contraceptive implants, before the woman is discharged from care. However, previous research has indicated that many midwives simply advise women to see their GP for contraceptive advice, thus missing a valuable opportunity to protect women from undesired repeat pregnancy before they are ready to have another child (3, 4). A recent survey of US midwives indicated that although 50% of midwives would welcome the opportunity to fit immediate post-partum contraception, fewer than 10% had ever done so (5)

References

1. Nursing and Midwifery Council, 2009. Standards for pre-registration midwifery education: p.69 [pdf]. Available at: [https://www.nmc.org.uk/globalassets/sitedocuments/standards/nmc-standards-for-preregistration-midwifery-education.pdf](https://www.nmc.org.uk/globalassets/sitedocuments/standards/nmc-standards-for-preregistration-midwifery-education.pdf%20)
2. 2. NICE 2006. Postnatal care up to 8 weeks after birth (CG37). <nice.org.uk/guidance/cg37>
3. Walker, S.H. & Davis, G., 2014. Knowledge and Reported Confidence of Final Year Midwifery Students Regarding Giving Advice on Contraception and Sexual Health. *Midwifery,* 30, e169-e176. Available at; [<http://dx.doi.org/10.1016/j.midw.2014.02.002>](http://dx.doi.org/10.1016/j.midw.2014.02.002)
4. McCance, K. & Cameron, S. 2014. Midwives' experiences and views of giving postpartum contraceptive advice and providing long-acting reversible contraception: a qualitative study. *Journal of Family Planning and Reproductive Health Care* Vol. 40, Iss. 3, pp. 177-183
5. Moniz, M., Roosevelt, L., Crissman, H., Kobernik, Dalton, V., Heisler, M., Low, L. 2017. Immediate Postpartum Contraception: A Survey Needs Assessment of a National Sample of Midwives. *Journal of Midwifery &Women’s Health* Volume 62, Issue 5. September/October 2017 pp.538–544

# SCIENTIFIC JUSTIFICATION

Women who deliver a baby will, on average, ovulate at 28 days after their delivery date (1, 2). Given that sperm can survive 5-7 days after intercourse, it is recommended that women start a reliable method of contraception by the time the baby is 21 days old (3,4). At present, although contraceptive advice is included as a topic to be covered during postnatal discharge (5), it is generally brief and often takes the form of advising women to contact their GP for contraception (6) Most women delay this visit until after Day 21, and so, if intercourse is resumed, are at risk of an unplanned pregnancy with a short inter-pregnancy interval. A UK study indicated that 7% of women giving birth and 6.4% of women requesting abortion had conceived less than a year after a previous delivery (7) Shorter inter-pregnancy intervals are riskier to mother and baby (8) and an unplanned, rapid second pregnancy may be a cause of maternal distress.

To encourage women to consider and to establish contraception before they are at risk of pregnancy many studies have looked at whether contraceptive advice could be effectively provided during maternity care (7, 9, 10, 11, 12) and some studies have examined at the outcomes of providing long-acting contraceptive methods during the post-natal period (13-18)

A recent study in a single hospital in the UK examined women’s views on post- natal provision of contraceptive care and found that 47% would prefer to have contraception provided before leaving the ward (19).

This project builds on previous studies by addressing the issues of postnatal provision of contraceptive care, but does so in a specific geographical and professional context. This project will look at the acceptability, and practical feasibility of provision of contraceptive care by midwives (not junior doctors or obstetricians). It will examine this is a specifically UK context, in the geographical area of the East of England, involving all seven acute trusts providing maternity care in this region. Health Education England (East of England) who are responsible for education of health care staff funds the project, and so the information derived from attitudes in this specific region may provide evidence, which will influence the educational provision for midwifery staff.

References

1. FSRH 2009 Postnatal Sexual and Reproductive Health Clinical Effectiveness Unit September 2009

2. Jackson, E. and Glasier, A. (2011) ‘Return of Ovulation and Menses in Postpartum Nonlactating Women’, Obstetrics & Gynecology, 117(3), pp. 657–662. doi: 10.1097/AOG.0b013e31820ce18c.

3. FSRH 2017 Contraception after pregnancy. Clinical Effectiveness Unit.

4. Speroff L, Mishell DR. 2008 The postpartum visit: it’s time for a change in order to optimally initiate contraception. Contraception 2008;78:90–98.

5. NICE 2015. Postnatal care up to 8 weeks after birth. Clinical guideline [CG37] Published date: July 2006 Last updated: February 2015 https://www.nice.org.uk/guidance/cg37/chapter/1-Recommendations

6. Walker, S. and Davis, G. (2014) ‘Views of final-year student midwives on giving postpartum contraception and sexual health advice’, Journal of Family Planning and Reproductive Health Care, 40(4). doi: 10.1136/jfprhc-2014-101028.

7. Heller R, Cameron S, Briggs R, et al. 2016. Postpartum contraception: a missed opportunity to prevent unintended pregnancy and short inter-pregnancy intervals. J Fam Plann Reprod Health Care 2016;42:93–98.)

8. Bigelow, C. A. and Bryant, A. S. (2015) ‘Short Interpregnancy Intervals’, Obstetrical & Gynecological Survey, 70(7), pp. 458–464. doi: 10.1097/OGX.0000000000000195.

9. Glasier AF, Logan J, McGlew TJ. 1996 Who gives advice about postpartum contraception? Contraception 1996;53:217–220.

10. Lopez LM, Grey TW, Chen M, et al. 2014. Strategies for improving postpartum contraceptive use: evidence from non-randomized studies. Cochrane Database Syst Rev 2014;11:CD011298.

11. Lopez LM, Hiller JE, Grimes DA, et al. 2010.Education for contraceptive use by women after childbirth. Cochrane Database Syst Rev 2012;8:CD001863.

12. Smith KB, van der Spuy ZM, Cheng L, et al. 2002. Is postpartum contraceptive advice given antenatally of value? Contraception 2002;65:237–243.

13. Ogburn JAT, Espey E, Stonehocker J.2005. Barriers to intrauterine device insertion in postpartum women. Contraception 2005;72:426–429.

14. Cameron, S. T. et al. (2017) ‘Feasibility and acceptability of introducing routine antenatal contraceptive counselling and provision of contraception after delivery: the APPLES pilot evaluation.(Report)’, BJOG: An International Journal of Obstetrics and Gynaecology, 124(13), p. 2009. doi: 10.1111/1471-0528.14674.

15. Cameron S. 2014. Postabortal and postpartum contraception. Best Pract Res Clin Obstet Gynaecol 2014;28:871–880.

16. Aiken ARA, Aiken CEM, Trussell J, et al. 2015. Immediate postpartum provision of highly effective reversible contraception. BJOG 2015;122:1050–1051.

17. Goldthwaite LM, Shaw KA. 2015.Immediate postpartum provision of long-acting reversible contraception. Curr Opin Obstet Gynecol 2015;27:460–464.

18. Cohen, R. et al. (2016) ‘Twelve-month contraceptive continuation and repeat pregnancy among young mothers choosing postdelivery contraceptive implants or postplacental intrauterine devices’, Contraception. Elsevier, 93(2), pp. 178–183. doi: 10.1016/J.CONTRACEPTION.2015.10.001.

19. Thwaites, A. et al. (2018) ‘Immediate postnatal contraception: what women know and think’, BMJ Sexual & Reproductive Health.

.

# 4 RESEARCH AIM(S)

This research project aims to assess the views of women, who are receiving midwifery care in trusts in the East of England region, about having their midwives provide not only contraceptive advice, but also contraceptive methods, including the fitting of contraceptive implants before leaving midwifery care.

It also aims to assess the views of midwives and, Heads of Midwifery (HoMs), working in trusts in the region regarding the possibility and practicality of providing such a service.

The completion of this project will provide a snapshot of the acceptability and feasibility of midwives expanding their role to include post-natal contraceptive provision in the East of England.

**Research questions**

1. To what extent are post-natal women supportive of the idea of an enhanced provision of contraception by midwives in the post-natal period? What is the extent of interest in particular methods, of particular groups, in terms of age, and pregnancy intention, or contraceptive use before pregnancy?
2. What are the views, concerns, perceived benefits, and reported needs of women with regard to the enhanced provision of contraception by midwives in the post-natal period?
3. What are the perceived benefits, concerns, logistical barriers, and training needs expressed by midwives with regard to the feasibility of enhanced provision of contraception by midwives in the post-natal period?

# 5 STUDY DESIGN and METHODS of DATA COLLECTION AND DATA ANALYISIS

This project will use a concurrent mixed method approach, using both qualitative interview data and quantitative survey data.

## **Views of Women (Patient views)**

*Quantitative Survey*

To obtain a snapshot of the extent of interest in and desirability of midwives extending their role to include provision of contraceptive care, a pragmatic sample of, as a minimum 140 women (20 from each trust in East of England), will be invited to complete brief highly focussed survey. This will provide key demographics (age, future pregnancy intention, previous contraception) and five items assessing interest in more (1)detailed advice, (2)prescription of progesterone only pill, (3) administration of contraceptive injection, (4) insertion of contraceptive implant and (5) insertion of intra-uterine contraception, at the time of post-natal discharge (or another time judged convenient by trust staff). Every woman will be offered the opportunity to take part until a minimum of 20 women have accepted, however in busy units we expect the final response to be higher. To limit unnecessary recruitment a maximum sample of 50 women from each acute unit will bring recruitment to an end.

*Analysis*

The results of this survey will be analysed descriptively to ascertain the extent to which women are interested in the provision of contraceptive methods by midwives. Analysis will identify subgroups of women who express more or less interest in this service, and the particular types of contraceptive provision that are of most interest to post-natal women. The free text comments will provide more detail in terms of the quantitative responses.

*Qualitative interviews*

Women will be asked to indicate whether they are willing to be interviewed on the topic of receiving contraceptive provision from midwives as part of an extended role. Their ideas, concerns and expectation will be explored in some depth, to provide qualitative data, which will contextualise and strengthen the quantitative findings. It is expected that these interviews will be carried out by telephone.

We will interview 4 women from each trust or acute unit, resulting in a total qualitative sample of 32 patient views. (One trust (East Suffolk & North Essex trusts comprises 2 acute units which will act as two separate sites)

*Analysis*

Thematic analysis of the views of women, and of midwives will be separately undertaken. This method of analysis will provide an overview of common themes expressed by each group. NVivo software will be used to facilitate thematic analysis.

In the analysis of interviews with women emphasis will be on identifying perceived need, perceived benefit and any concerns or barriers expressed. Unanticipated issues may arise from the interviews that have not been previously identified.

(For Flow Diagram of patient participation, see Appendix1)

## **Staff Views**

*Qualitative interviews* with midwives and HoMs will provide data regarding the opinions, feelings, concerns, attitudes and experiences of staff with regard to taking on an extended role in contraceptive provision. A pragmatic sample of 32 midwives, from each of the seven trusts providing midwifery care in the EoE region, will be used, to enable a cross–section of midwives in the region to be included. (In one trust, in which there are two separate units, 4 staff from each acute unit may be interviewed, resulting in a maximum of 32 staff). Heads of midwifery from each trust will also be invited to take part to ascertain higher, strategic management views.)

Analysis of interviews with midwives will focus on their views on the benefits for the women in their care, the logistical and practical barriers to instituting the service in their place of work, educational needs, professional role perception and key barriers and concerns. Some comparison between the views of women and the views of midwives will be carried out.

### Inclusion/Exclusion

Midwives will be asked to exclude any women who they deem to have experienced a traumatic or distressing birth experience or pregnancy, in order to minimise any further distress for these women. A list of conditions that are considered as exclusion criteria, due to their traumatic or distressing nature, is provided at the end of this protocol.

Due to lack of funding for translation services, women who do not speak English well enough to understand the participant consent forms, or do not read English, will be excluded from the study.

Anyone deemed by the midwives caring for them, to lack capacity to understand the nature of the research, and to give informed consent, due to a learning difficulty, will be excluded from the research.

All participants will be aged 16 years or above.

### Consent

Women will be provided with the survey, and participant information on the project, and given time to consider whether to take part. To facilitate consideration of whether or not to take part, the surveys will be collected either in person by the midwives, or posted in a secure, locked box, or electronically by submitting an online version of the survey. Consent to participation will be indicated by returning the survey, and this will be clearly indicated on the survey. There will be no separate written consent for the survey.

Women who indicate interest in taking part in a telephone interview on the topic will be sent participant information and consent forms by email or post prior to the interview. A pre-paid envelope will be provide to allow women to return the consent form, after which a telephone interview will take place.

Midwives and Heads of Midwifery will be sent similar participant information and participant consent forms prior to taking part. Where interviews are conducted in person, written consent will be obtained, but where interviews are conducted by telephone, verbal consent will be recorded using the questions on the consent form, which will be read out at the start of the interview.

### Withdrawal of consent

It will not be possible to withdraw consent from inclusion in the survey once the survey has been returned, since participant identifiable information will not be included. This will be clearly stated in the information accompanying the survey.

Interviews will have the right to withdraw consent after participation up to 30 days after the interview. They will be provided with the contact details of the lead researcher (work phone and email) and also with a withdrawal slip, which can be completed and posted. It will be made clear that no explanation need be provided.

# 6 STUDY SETTING

The study is a multi-centred study. It will take place in the seven acute trusts providing maternity care in the East of England.

**7 SAMPLE AND RECRUITMENT**

**7.1 Eligibility Criteria**

Women will be recruited who are receiving post-natal care in the acute trusts where the study is situated. Women will be asked to complete the survey in the first 10 days after delivery, either before discharge from the ward, or prior to discharge from community maternity services.

Midwifery staff will be recruited from the 7 acute trusts.

**7.1.1 Inclusion criteria**

**Aged 16 or above**

**Receiving postnatal care after an uncomplicated delivery**

**Able to read English**

**Having capacity to consent**

**7.1.2 Exclusion criteria**

**Women experiencing a distressing or traumatic birth** - Midwives will be asked to exclude any women who they deem to have experienced a traumatic or distressing birth experience or pregnancy, in order to minimise any further distress for these women. A list of conditions deemed as traumatic or distressing is provided at the end of the protocol.

**Women who cannot read or write English** - Due to lack of funding for translation services, women who do not speak English well enough to understand the participant consent forms, or do not read English, will be excluded from the study.

**Women lacking capacity to consent** - Anyone deemed by the midwives caring for them, to lack capacity to understand the nature of the research, and to give informed consent, due to a learning difficulty, will be excluded from the research.

**Women under the age of 16 years.**

**7.2 Sampling**

**7.2.1 Size of sample**

### Justification of Proposed Samples

*Survey Respondents;*

No inferential analysis will be carried out so no formal power calculation has been attempted for the quantitative sample.

Given the homogeneous nature of women using maternity services in this small geographical area, a combined sample of at least 140 survey respondents is sufficient to provide the descriptive data needed to explore the interest in enhanced contraceptive provision, and to explore differences between subgroups of women. In busier units we expect that the response rate to the sample will be higher. To limit the burden on patients and staff we will stop distribution of surveys once a maximum of 50 have been returned from any one acute unit. This will result in a minimum of 140 (20 women x 7 acute trusts)and a maximum of 400 (50 women x 8 acute units) survey respondents. (One trust (East Suffolk North Essex) comprises two acute units, which will act as two separate sites)

*Qualitative interviewees;*

A sample of 32 women is of sufficient size to allow data saturation from a thematic analysis of their views expressed at interview.

Similarly, a sample of 24 midwives is sufficient to allow such data saturation, and 7-8 Head of midwifery will provide a strategic overview for each acute unit.

**7.2.2 Sampling technique**

### 7.3 Recruitment & Consent:

Women will be recruited to complete a short survey by community or ward-based midwifery staff (including research midwives), already involved in their care. Whilst this ensures that women are not approached in person by a researcher during their care, it raises the possibility that women may feel obliged to take part. The information given on the survey, and the approach the recruiting midwives will take, will stress the voluntary nature of participation, and the fact that the choice to take part or not take part will have no impact on care.

Women will be provided with information on the survey and given time to consider whether to take part. To facilitate time to consider whether to take part, the surveys will be collected either in person by the midwifery staff, or by return to a secure, locked post box on the ward, or online at a time of the participant’s convenience.

In one trust (CUHNFT) staff have indicated that the survey should be distributed online, via a patient engagement website Rosie Maternity Voices. If this is unsuccessful, surveys will be distributed by midwifery staff, and returned as above.

The surveys will contain a section where the woman can volunteer to take part in a further telephone interview. Again it will be made very clear that this is optional, and the women will be contacted initially to arrange a convenient time, and to answer any queries. The telephone interview will then be conducted subsequently at the arranged time. Further participant information and consent forms will be emailed or posted prior to the interview by the research team. A pre-paid envelope to allow return of the consent form will be provided. Once this has been received, the research team will conduct a telephone interview.

Midwives will be recruited by direct approach through gatekeepers, i.e. managers, university link lecturers, by email or in person. They will also be alerted to the study by posters in staff areas.

**8.3 Peer review**

This study has been reviewed by the funder and by an independent researcher within Anglia Ruskin University

###

Exclusion Criteria –

**Exclude due to “traumatic or distressing pregnancy or birth”**

Women who have had a stillbirth.

Women whose neonate has died.

Women whose babies have a congenital abnormality.

Women whose babies are sufficiently unwell to be cared for off the post-natal ward (e.g. in NICU).

Women who are planning to have their baby adopted or fostered voluntarily, or because of acting as a surrogate, or involuntarily due to statutory order/social services intervention.

Women whose pregnancy is known to be the result of rape.

Women experiencing puerperal psychosis, or currently unwell due to schizophrenia, bipolar disorder, psychotic depression.

Women who have had an unplanned Category 1 caesarean section (i.e. carried out due to immediate threat to the life of the woman or fetus).

Women who have experienced an ante-partum or post-partum haemorrhage sufficient to require blood transfusion.

Women who have experienced an intervention, which has caused them to become infertile e.g. emergency hysterectomy.

Women who have been diagnosed with HIV during their pregnancy.

Women who have experienced loss of a partner due to bereavement during their pregnancy.

Women who are, for an unanticipated reason, distressed during their postnatal stay, such that midwifery staff feel an invitation to take part in the research would cause additional distress.

**Additional exclusion criteria (unrelated to nature of pregnancy)**

Women who have a learning difficulty.

Women who do not read or write English well enough to complete the survey unaided.

Women under 16 years of age

| Screening  T:0 |
| --- |
| Recruitment  & Survey  distribution  T:1 |
| Survey Collection  T:2 |
| Recruitment to Interview  T:3 |
| Interviews carried out  T:4 |

###

Member of research team carries out interview

Woman delivers baby under care of acute trust

After delivery Midwifery team assess if eligible for inclusion in survey

Before final discharge from midwifery care - woman is given PIS and survey by midwifery staff

Either T1a or T1b – according to working practices of each acute unit

After having time to consider whether or not to take part – women completes survey either online or on paper.

T1b – Day 5 in community

T1a – on post-natal ward

Exclusion Criteria

-Having experienced a traumatic or distressing pregnancy and/or birth (see p.11 above).

-Not reading English sufficiently well to complete the survey unaided.

-Lacking capacity to consent to taking part in the research.

-Being under 16 years of age.

Inclusion Criteria

-Attending a trust in the East of England region for maternity care.

-Reads English sufficiently well to complete survey unaided.

-Aged 16 years or over.

Survey submitted online

OR

Paper copy collected by midwifery staff on ward or in community or returned in secure, locked ‘post’ box

OR

If requested stamped, pre-addressed envelope given to post survey

Excluded

Woman decides whether to give contact details for interview in text box at end of survey

NO

YES
