# Supplementary material for: The views of postnatal women and midwives on midwives providing contraceptive advice and methods: a mixed method concurrent study
Source: BMC Pregnancy Childbirth. 2021 Jun 2;21:411. doi: 10.1186/s12884-021-03895-2 (PMC8170056; doi:10.1186/s12884-021-03895-2)
Supplement: Supplementary file 4 — Additional file 4. [file 12884_2021_3895_MOESM4_ESM.docx]

Exclusion Criteria –

**Exclude due to “traumatic or distressing pregnancy or birth”**

Women who have had a stillbirth.

Women whose neonate has died.

Women whose babies have a congenital abnormality.

Women whose babies are sufficiently unwell to be cared for off the post-natal ward (e.g. in NICU).

Women who are planning to have their baby adopted or fostered voluntarily, or because of acting as a surrogate, or involuntarily due to statutory order/social services intervention.

Women whose pregnancy is known to be the result of rape.

Women experiencing puerperal psychosis, or currently unwell due to schizophrenia, bipolar disorder, psychotic depression.

Women who have had an unplanned Category 1 caesarean section (i.e. carried out due to immediate threat to the life of the woman or fetus).

Women who have experienced an ante-partum or post-partum haemorrhage sufficient to require blood transfusion.

Women who have experienced an intervention which has caused them to become infertile e.g. emergency hysterectomy.

Women who have been diagnosed with HIV during their pregnancy.

Women who have experienced loss of a partner due to bereavement during their pregnancy.

Women who are, for an unanticipated reason, distressed during their postnatal stay, such that midwifery staff feel an invitation to take part in the research would cause additional distress.

**Additional exclusion criteria (unrelated to nature of pregnancy)**

Women who have a learning difficulty.

Women who do not read or write English well enough to complete the survey unaided.

Women under 16 years of age
