# Supplementary material for: The views of postnatal women and midwives on midwives providing contraceptive advice and methods: a mixed method concurrent study
Source: BMC Pregnancy Childbirth. 2021 Jun 2;21:411. doi: 10.1186/s12884-021-03895-2 (PMC8170056; doi:10.1186/s12884-021-03895-2)
Supplement: Supplementary file 5 — Additional file 5. [file 12884_2021_3895_MOESM5_ESM.docx]

Draft Interview Schedule for Women

Check Consent.

Demographics – age, age of baby, family complete?

What are your plans for contraception now that the baby is born?

Had you been using contraception prior to becoming pregnant? Which method?

Would it have been helpful to you if your midwife could have started you on a method/ your method?

What would be the advantages of this for you?

How could it be arranged to make it most convenient for you?

Would you have any concerns about it?

What factors would make you reluctant to receive advice or a method from your midwife?

Would there be any methods you would not want the midwife to supply?

When would be the best time to hear advice about contraception? Before the baby is born, on discharge, at Day 5?

Would you have been prepared to return to the unit to have an implant or coil fitted, if that was what you had decided you wanted?

Any other views or comments?
